# Supplementary material for: High-throughput screening identifies cell cycle-associated signaling cascades that regulate a multienzyme glucosome assembly in human cells
Source: PLoS One. 2023 Aug 4;18(8):e0289707. doi: 10.1371/journal.pone.0289707 (PMC10403072; doi:10.1371/journal.pone.0289707)
Supplement: S1 Fig — (PDF) [file pone.0289707.s001.pdf]

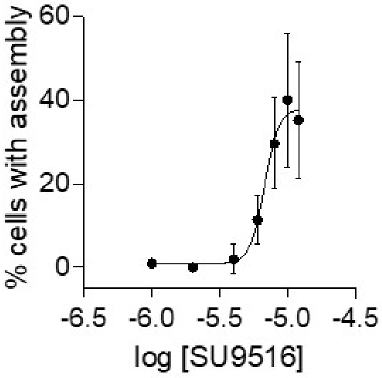

S1 Fig. EC_{50} measurement of SU9516. HeLa-T-PFK1G cells were treated with SU9516 in titration for 25 hours and the number of cells showing PFK1 assemblies was assessed. Error bars represent standard deviations of at least three independent trials. At least 300 cells were analyzed per condition.
