# Supplementary material for: High-throughput screening identifies cell cycle-associated signaling cascades that regulate a multienzyme glucosome assembly in human cells
Source: PLoS One. 2023 Aug 4;18(8):e0289707. doi: 10.1371/journal.pone.0289707 (PMC10403072; doi:10.1371/journal.pone.0289707)
Supplement: S2 Fig — (PDF) [file pone.0289707.s002.pdf]

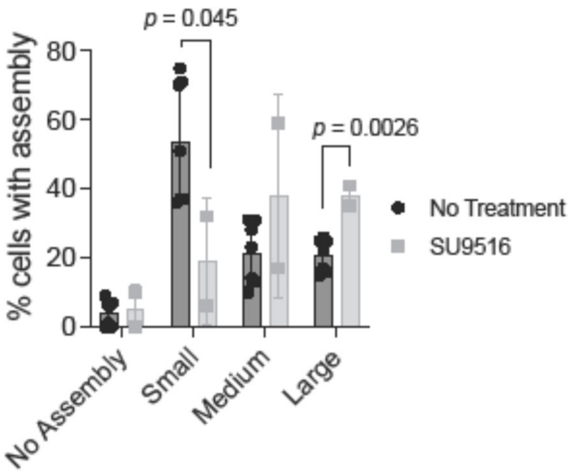

S2 Fig. **Effect of SU9516 on transiently expressed PFK1-mEGFP in HeLa cells.** HeLa cells transiently expressing PFK1-mEGFP were treated with 57.5 µM SU9516 for 5 hours. Following treatment, the percent of cell population containing no assembly, small, medium, or large sized assemblies were assessed. Error bars represent standard errors. Statistical significance was determined using student's two-tailed t test.
