# Supplementary material for: High-throughput screening identifies cell cycle-associated signaling cascades that regulate a multienzyme glucosome assembly in human cells
Source: PLoS One. 2023 Aug 4;18(8):e0289707. doi: 10.1371/journal.pone.0289707 (PMC10403072; doi:10.1371/journal.pone.0289707)
Supplement: S3 Fig — (PDF) [file pone.0289707.s003.pdf]

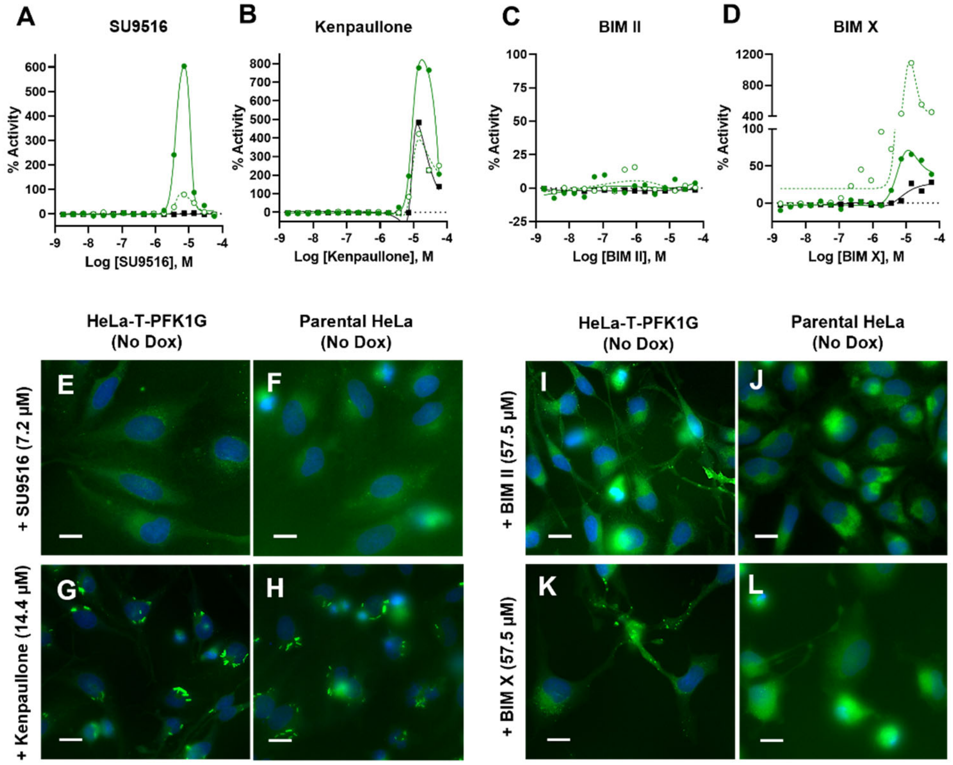

S3 Fig. Formation of artifactual fluorescent puncta by chemical compounds. (**A-D**) Formation of artifactual fluorescent puncta in the presence of SU9516 (**A**), kenpaullone (**B**), BIM II (**C**) or BIM X (**D**) were quantified by an ImageXpress Confocal HT.ai (Molecular Devices) from HeLa-T-PFK1G cells with doxycycline induction (●, solid green circles) or without doxycycline (○, open green circles), and also from parental HeLa cells (■, black squares). Data from HeLa-T-PFK1G cells with or without doxycycline induction were then normalized to DMSO as 0% activity and 11.6 µM SU9516 as 100% activity; while data from parental HeLa cells (■, black squares) were normalized to DMSO as 0% activity and 11.6 µM kenpaullone as 100% activity. GraphPad Prism was used to fit with bell-shaped curves as a function of compound concentration. (**E-L**) Representative images from an ImageXpress imager in the presence of an indicated compound from HeLa-T-PFK1G cells without doxycycline induction (**E**, **G**, **I** and **K**) and from parental HeLa cells (**F**, **H**, **J** and **L**). Note that BIM II and BIM X are control compounds known to cause artifactual fluorescent responses (24). Scale bar, 20 µm.
