# Supplementary material for: High-throughput screening identifies cell cycle-associated signaling cascades that regulate a multienzyme glucosome assembly in human cells
Source: PLoS One. 2023 Aug 4;18(8):e0289707. doi: 10.1371/journal.pone.0289707 (PMC10403072; doi:10.1371/journal.pone.0289707)
Supplement: S1 Table — (PDF) [file pone.0289707.s004.pdf]

S1 Table. Initial 16 hit compounds from the LOPAC screen.

NCGC ID	Compound Name
NCGC00013043-08	1,10-Phenanthroline monohydrate
NCGC00015856-07	Prochlorperazine
NCGC00014483-11	Nortriptyline hydrochloride
NCGC00015233-04	Calmidazolium chloride
NCGC00015281-03	Clemastine fumarate
NCGC00015376-06	N,N-Dihexyl-2-(4-fluorophenyl)indole-3-acetamide
NCGC00016888-03	Fluoxetine hydrochloride
NCGC00015582-06	Kenpaullone
NCGC00016012-09	Promazine hydrochloride
NCGC00016012-09	Triflupromazine hydrochloride
NCGC00016888-03	S-(+)-Fluoxetine hydrochloride
NCGC00094244-06	SU 9516
NCGC00167785-02	PAC-1
NCGC00186031-01	ARP 101
NCGC00015701-05	DL-alpha-Methyl-p-tyrosine
NCGC00094144-06	L-alpha-Methyl-p-tyrosine
