# Supplementary material for: High-throughput screening identifies cell cycle-associated signaling cascades that regulate a multienzyme glucosome assembly in human cells
Source: PLoS One. 2023 Aug 4;18(8):e0289707. doi: 10.1371/journal.pone.0289707 (PMC10403072; doi:10.1371/journal.pone.0289707)
Supplement: S2 Table — (PDF) [file pone.0289707.s005.pdf]

S2 Table. References of the small molecules that induced PFK1-mEGFP assemblies from the kinase inhibitor-enriched library.

NCGC ID	Primary Target	Reference
NCGC00263213-01	IKK-2 (IKK-beta) Inhibitor	Sommers et al J Pharmacol Exp Ther 330 377-88 (2009)
NCGC00346518-01	Carbapenem Antibiotic	Pryka Ann Pharmacother 28 1045-54 (1994)
NCGC00348107-01	CSF1R (c-FMS) Inhibitor	USA Patent US7705042
NCGC00094087-06	Inosine 5'-Monophosphate Dehydrogenase (IMPDH) Inhibitors	Koyama et al Biochem Pharmacol 32 3547-53 (1983)
NCGC00159346-05	Fungal Squalene Monooxygenase Inhibitor	Ryder Clin Exp Dermatol 14 98-100 (1989)
NCGC00344512-01	Opioid receptor antagonist	Schmidhammer et al J Med Chem 32 418-21 (1989)
NCGC00167513-03	EGFR (HER1; erbB1) Inhibitor	Hennequin et al J Med Chem 45 1300-12 (2002)
NCGC00165811-03	Inhibitor of nuclear factor kappa b kinase subunit beta (IKK-2) Inhibitor	Onai et al Cardiovasc Res 63 51-9 (2004)
NCGC00346698-01	mTORC1/2 inhibitor	Pike et al Bioorg Med Chem Lett 23 1212-6 (2013)
NCGC00346747-02	NFkappaB-inducing kinase Inhibitor	USA Patent US2011086834
NCGC00347280-01	IKK-2 (IKK-beta) Inhibitor	Murata et al Bioorg Med Chem Lett 14 4019-22 (2004)
NCGC00241982-03	ROCK 1, ROCK 2 Inhibitor	Stavenger et al J Med Chem 50 2-5 (2007)
NCGC00346553-01	CDK7	Ali et al Cancer Res 69 6208-15(2009)
NCGC00263129-01	CDK4, 6	Fry et al Mol Cancer Ther 3 1427-38 (2004)
NCGC00018248-08	Cyclooxygenase-1/2 Inhibitor	Noble et al Drugs 51 424-30 (1996)
NCGC00166111-04	Estrogen Receptor (ER) Agonist (nuclear)	Clark et al J Anim Sci 49 46-65 (1979)
NCGC00181306-03	Tubulin depolymerization inhibitor	Plenta Semin Oncol 28 3-7 (2001)
NCGC00346893-01	MAPKAP-K1 (RSK; p90Rsk) Inhibitor	Smith et al Cancer Res 65 1027-34 (2005)
NCGC00346542-01	Aurora kinase inhibitor	Hauf et al Journal of Cell Biology 161 281-94 (2003)
NCGC00346652-01	Aurora kinase inhibitor	Jani et al Mol Cancer Ther 9 883-94 (2010)
NCGC00263125-01	mTORC1/2 inhibitor	Weinberg Anticancer Drugs 27 475-87 (2016)
NCGC00346681-01	PI3K Inhibitor	Chang et al Clin Cancer Res 15 7116-26 (2011)
NCGC00346959-01	Lck Kinase Inhibitors	Martin et al J Med Chem 49 4981-91 (2006)
NCGC00015420-09	Casein Kinase II (CK2) Inhibitor	Srinivas et al Med Res Rev 27 591-608 (2007)
