# Supplementary material for: High-throughput screening identifies cell cycle-associated signaling cascades that regulate a multienzyme glucosome assembly in human cells
Source: PLoS One. 2023 Aug 4;18(8):e0289707. doi: 10.1371/journal.pone.0289707 (PMC10403072; doi:10.1371/journal.pone.0289707)
Supplement: S1 Raw images — (PDF) [file pone.0289707.s006.pdf]

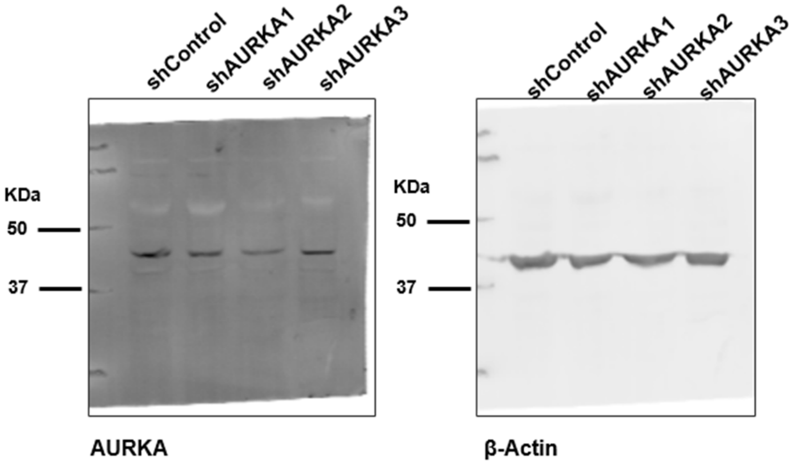

Western blot analysis of aurora kinase A (AURKA) from HeLa cells. Uncropped western blots of **Fig 5A** showing the knockdown of AURKA in HeLa cells.
